# Dynamic Alterations of Amplitude of Low-Frequency Fluctuations in Patients With Drug-Naïve First-Episode Early Onset Schizophrenia

**DOI:** 10.3389/fnins.2020.00901

**Published:** 2020-10-06

**Authors:** Qiang Li, Xiaohua Cao, Sha Liu, Zexuan Li, Yanfang Wang, Long Cheng, Chengxiang Yang, Yong Xu

**Affiliations:** ^1^Shanxi Key Laboratory of Artificial Intelligence Assisted Diagnosis and Treatment for Mental Disorder, First Hospital of Shanxi Medical University, Taiyuan, China; ^2^Department of Psychiatry, First Hospital/First Clinical Medical College of Shanxi Medical University, Taiyuan, China; ^3^Department of Mental Health, Shanxi Medical University, Taiyuan, China

**Keywords:** early onset schizophrenia, dynamic amplitude of low-frequency fluctuations, variability, middle temporal gyrus, default mode network

## Abstract

Abnormalities in static neural activity have been widely reported in early onset schizophrenia (EOS). However, dynamic brain activity alterations over time in EOS are unclear. Here, we investigated whether temporal dynamic changes in spontaneous neural activity are influenced by EOS. A total of 78 drug-naïve first-episode patients with EOS and 90 healthy controls (HCs) were enrolled in this study. Dynamic amplitude of low-frequency fluctuations (dALFF) was performed to examine the abnormal time-varying local neural activity in EOS. Furthermore, we investigated the relationships between abnormalities in dALFF variability and clinical characteristics in EOS patients. Compared to HCs, EOS patients showed significantly decreased dALFF variability in the bilateral precuneus, right superior marginal gyrus, right post-central gyrus and increased dALFF in the right middle temporal gyrus (MTG). Moreover, increased dALFF variability in MTG was negatively associated with negative symptoms in EOS. Our findings reveal increased dynamic local neural activity in higher order networks of the cortex, suggesting that enhanced spontaneous brain activity may be a predominant neural marker for brain maturation. In addition, decreased dALFF variability in the default mode network (DMN) and limbic system may reflect unusually dynamic neural activity. This dysfunctional brain activity could distinguish between patients and HCs and deepen our understanding of the pathophysiological mechanisms of EOS.

## Introduction

Early onset schizophrenia (EOS) is defined as schizophrenia during adolescence or before adolescence, and is associated with severe impairments including hallucinations, delusions and cognitive deficiencies (Kyriakopoulos et al., 2008; Paus et al., 2008; Jiang et al., 2015). EOS patients are less effected by environment and medication compared with adult schizophrenia, which provides a unique opportunity to explore the pathophysiological mechanisms of schizophrenia (Vyas et al., 2010).

Resting-state functional magnetic resonance imaging (rs-fMRI) is a brain imaging technique for investigating brain oscillatory modulations, functional integration and brain network abnormalities by capturing different blood oxygenation level-dependent (BOLD) signals (Lowe, 2012; Zheng et al., 2016; Wang et al., 2019c). It can be a particularly useful tool for examining the neural bases of psychopathologies and for investigating differences between EOS patients and healthy controls (HCs). Recently, abnormal brain functional integration have been observed in EOS, including significantly reduced functional activity in the prefrontal cortex (PFC), anterior cingulate cortex (ACC), frontal operculum and decreased neural activity of PFC (Yang et al., 2014; Jiang et al., 2015; Li et al., 2015). In particular, these functional disruptions were correlated with dysfunctional working memory (WM) performance (Kyriakopoulos et al., 2012; Bittner et al., 2015). Additionally, functional connectivity (FC) studies have demonstrated hyperconnectivity between the medial frontal gyrus and areas of default mode network (DMN) (Tang et al., 2013), as well as disrupted connectivity in ACC, limbic, cerebellum, temporal lobe, post-central gyrus, and early visual cortex networks. Moreover, abnormal FC patterns in higher order cortical networks have been shown to be significantly correlated to severe psychiatric symptoms and cognitive impairments (White et al., 2011; Bittner et al., 2015; Jiang et al., 2015; Li et al., 2015). Such abnormal brain functional activity might also contribute to the neuropathological mechanisms of EOS, potentially making abnormal functional activity an early biomarker for the early diagnosis of EOS.

Early onset schizophrenia is considered to be an abnormal developmental disease manifesting as abnormal neural activity. To characterize the spontaneous neural activity of the brain, the amplitude of low-frequency fluctuations (ALFF) method was developed to study abnormal neural activity in brain-related disorders (Cakir, 2019; Wu et al., 2019; Wang et al., 2020a,b). Recently, ALFF has also been used to explore abnormal neural activity in EOS, revealing increased ALFF in the orbitofrontal cortex, caudate body, temporal, parietal lobule, as well as decreased ALFF in the ventral precuneus and cerebellum (Zheng et al., 2016; Liang et al., 2019). According to previous findings, ALFF is an established approach to characterizing spontaneous neural activity and effectively delineating the potential pathophysiological mechanisms of EOS. However, most of the aforementioned studies rely on the hypothesis that neural activity remains stationary during fMRI scanning (Cui et al., 2019; Chen et al., 2020), while, in fact, human neural activity is dynamic and correlated to ongoing rhythmic activity over time (Hutchison et al., 2013; Calhoun et al., 2014; Li et al., 2018). Recently, abnormal dynamic spontaneous neural activity has been identified in adult schizophrenia (Shen et al., 2014; Hare et al., 2017; Zhang et al., 2019b), but the nature of the contribution of abnormal dynamic neural activity to the onset of EOS is poorly understood.

In the current study, we examined the dynamic neural activity in patients with EOS, and used a combined approach of ALFF and “sliding windows” to reveal differences in brain activity patterns across EOS patients and HCs. A total of 78 untreated EOS and 90 HCs were recruited in this study. First, we applied dynamic ALFF (dALFF) to reveal potential changes in spontaneous neural activity patterns. Next, we identified the brain areas with abnormal dALFF variability. Finally, we used correlation analyses to reveal the relationships between abnormal dynamic neural activity and clinical symptomatology in EOS; these analyses may be used as neurodevelopmental evidence to distinguish patients with EOS from HCs.

## Materials and Methods

### Participants

We recruited 78 patients with EOS (age range: 9–17.9 years) and 90 age, sex, and education matched HCs (age range: 7.5–17.9 years) to participate in this study. All of the patients were recruited from January 2010 to April 2019 in the Department of Psychiatry at the First Hospital of Shanxi Medical University, Shanxi China. All of the patients were independently assessed by two experienced psychiatrists based on the Structured Clinical Interview for DSM-IV-TR, patient version (SCID-I/P). In addition, all of the patients were interviewed 6 months following the termination of the study to confirm a final diagnosis of schizophrenia. Clinical symptoms in patients were assessed using the Positive and Negative Syndrome Scale (PANSS). All of the patients were in their first episode and were drug-naïve prior to scanning. Inclusion criteria for patients were as follows: (1) no-morbid Axis-I or Axis-II diagnoses and (2) duration of illness <1 year.

All of the HCs were enrolled from the local community through advertisements and were excluded on the basis of (1) any past or current neurological disorders or first-degree relatives history of hereditary neurological disorders; (2) history of head injury with loss of consciousness; (3) alcohol or substance abuse; (4) MRI contraindications; and (5) incompatible implants; All of the participants provided informed written consent, and the study was approved by the Ethics Committee of the First Hospital of Shanxi Medical University.

### MRI Data Acquisition

MRI data were obtained with a Siemens Trio 3.0 Tesla scanner (Erlangen, Germany). Participants were instructed to stay awake with their eyes closed, and not to fall asleep or move during the scanning procedure. Finally, no participants were excluded due to falling asleep or opening their eyes. Functional images were collected using an echo-planar imaging (EPI) sequence with the following parameters: repetition time (TR) = 2500 ms; echo time (TE) = 30 ms; matrix = 64 × 64, 32 axial slices; slice thickness = 3 mm with 1 mm gap; flip angle = 90°; field of view = 240 × 240 mm^2^; voxel size = 3.75 × 3.75 × 4 mm^3^; and 212 volumes.

### Functional MRI Data Preprocessing

Rest-state functional MRI data preprocessing was carried out by using DPABI^1^ software toolbox. For each participant, the first 10 functional volumes were discarded to ensure the equilibration of the magnetic field. Subsequently, slice timing and realignment correction were performed to correct temporal differences between slices and head motion between time points. Consequently, six EOS patients and 11 HCs were excluded due to head motion exceeding 2.5 mm or 2.5° in any direction; thus, 72 EOS patients and 79 HCs were finally included in this study. The remaining images were further normalized into standard stereotactic EPI template in Montreal Neurological Institute (MNI) space and resampled to a 3 × 3 × 3 mm^3^ resolution. The normalized images were linearly detrended and nuisance covariates including Friston 24 motion parameters (Friston et al., 1996), white matter signal, cerebrospinal fluid signal and global signal were regressed out by the multiple regression model to reduce the effects of signal drifts and non-neuronal BLOD fluctuations (Tomasi and Volkow, 2012). Subsequently, data were subjected to band-pass temporal filtering (0.01–0.08 Hz). In addition, the mean frame-wise displacement (FD), which indexes volume-to-volume alterations in head position (excluding any volume with a mean FD value exceeding 0.5 mm), was calculated from derivatives of the rigid body realignment estimates, and further assesses the confounding effect of head motion on statistical analyses (Power et al., 2012; Wang et al., 2019b). Finally, all of the data were smoothed with an 8 mm isotropic Gaussian kernel for statistical analyses.

### Dynamic Amplitude of Low-Frequency Fluctuations Analysis

The dALFF was calculated by using a Dynamic BC toolbox (Liao et al., 2014). Based on the processed data, the dALFF calculation was constrained to the whole brain mask. The sliding window method was performed to characterize the dynamic neural activity in this study. Previous research has suggested that the window length is a critical parameter to acquire dynamic spontaneous brain activity (Li et al., 2018; Cui et al., 2019), in which too short a window length might not allow for sufficient estimation of dynamic changes, and too long a window length may not be sufficiently sensitive to detect dynamic activity (Zhang et al., 2019a). Therefore, to calculate the dALFF of each participant, we applied 50 TR (125 s) as a moderate sliding window length and 5 TR (10 s) as the step size (Li et al., 2018; Cui et al., 2019; Yao et al., 2020). The post-processed 202 volume of each participant was divided into 31 windows, and the ALFF map was computed within each window. Additional window lengths of 30 TR (75 s) and 80 TR (200 s) were also calculated to verify our findings. Subsequently, to evaluate the temporal variability of dALFF, we calculated the variance of ALFF maps of each participant by using standard deviation (SD). Finally, for all subjects, the dALFF variability was then transformed into standardized z-scores by subtracting the mean and dividing by the SD across each voxel to enhance date normality. In addition, the static ALFF (sALFF) was further calculated and transformed into standardized z-scores for each subject to check if abnormal sALFF and dALFF assumed similar spatial distribution and offered additional clues to the etiology of EOS.

### Statistical Analyses

The dALFF variability distribution in both groups was obtained by averaging dALFF values at each voxel across subjects within EOS and HCs groups. Two-sample *t*-tests were performed to investigate in dALFF variability group differences. Age, sex and mean FD were regressed as covariates. We applied a Gaussian Random-Field (GRF) method (voxel level, *p* < 0.005; cluster level, *p* < 0.05) for multiple comparison correction. Similarly, two-sample *t*-tests were also applied to probe group differences in sALFF between patients and HCs using the same covariates and GRF correction method. We then extracted the mean dALFF (sALFF) values of each cluster as ROIs to conduct a *post hoc* comparison (*p* < 0.05, Bonferroni correction) and further investigated the potential correlations of abnormal dynamic (static) neural activity and symptom severity of patients with EOS. We then used Spearman’s correlation analyses to test the relationship between abnormal dALFF (sALFF) variability and PANSS score of EOS patients.

## Results

### Participants and Demographic Characteristics

The demographic and clinical features of all the participants are presented in Table 1. No significant difference in age (Mann-Whitney *U* test, *p* = 0.4591) and sex (*χ^2^* test, *p* = 0.2853) were found between EOS and HCs, but we observed a significant difference in mean FD (*p* = 0.0008) between the two groups.

**TABLE 1:** Demographic data of early-onset schizophrenic (EOS) and healthy controls (HCs).

Characteristic	EOS	HC	*p* value
	
	Mean ± SD	Mean ± SD	
Sample size	72	79	
Age (years)	14.7 ± 1.78	14.3 ± 2.17	0.4591^a^
Sex (F/M)	48/24	46/33	0.2853^b^
Handedness	72\0	79\0	
Mean FD	0.12 ± 0.10	0.15 ± 0.08	0.0008^a^
Total score (PANSS)	68.9 ± 18.4		
Positive score	15.8 ± 5.19		
Negative score	16.3 ± 7.53		
General	32.9 ± 8.58		

*FD, frame-wise displacement; PANSS, Positive and Negative Syndrome Scale; Mean ± SD, values are mean ± standard deviation; F/M, Female/Male.^*a*^ Mann-Whitney *U*-test.^*b*^ Chi-square test.*

### Group Differences in dALFF (sALFF) Between Patients With EOS and HCs

**FIGURE 1:**
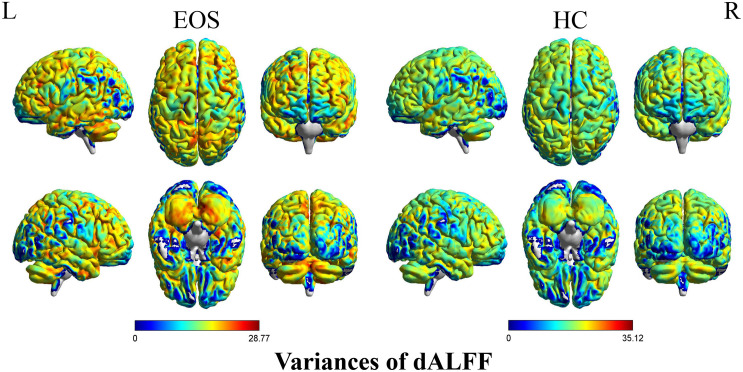
Pattern of dALFF variability in early onset schizophrenia (EOS) patients and healthy controls (HCs). EOS patients and HCs exhibited significant differences in the spatial distribution of dALFF variability. dALFF, dynamic amplitude of low-frequency fluctuations; HCs, healthy controls; EOS, early onset schizophrenia; L, left; R, right.

**FIGURE 2:**
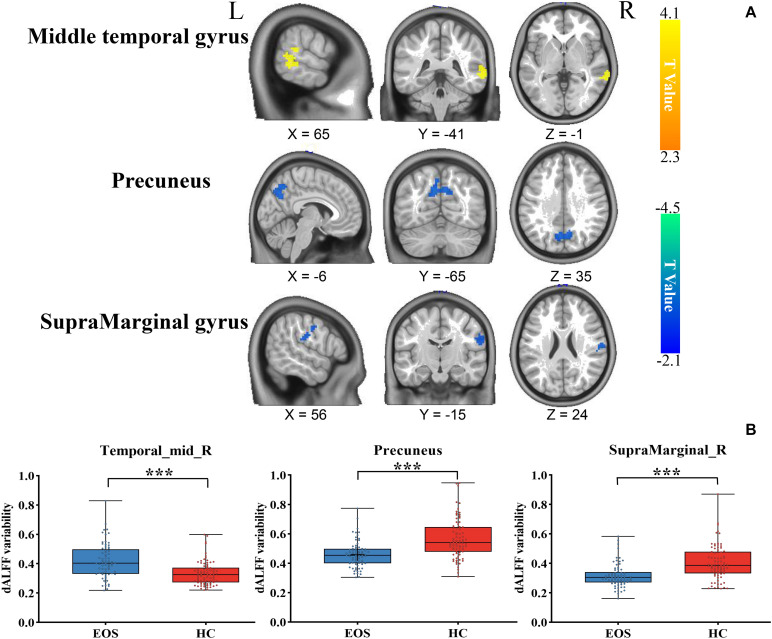
Brain region differences in dALFF variability in EOS compared to HCs. **(A)** EOS showed increased dALFF variability in the right middle temporal gyrus (MTG) (winter color) and decreased dALFF variability in the bilateral precuneus, right supramarginal gyrus and post-central gyrus compared to HCs (warm color) (*GRF* corrected; *p* < 0.005; cluster level, *p* < 0.05; minimum clusters size of 20 voxels). **(B)** The graph exhibiting the ROI-wise *post hoc* comparison results. *p < 0.01, uncorrected; ***p < 0.0001, Bonferroni correction.

Early onset schizophrenia patients exhibited different spatial distribution of dALFF variability, compared to HCs (Figure 1). Specifically, EOS patients displayed significantly reduced dALFF variability in the bilateral precuneus, right supramarginal gyrus and post-central gyrus and increased dALFF variability in the right middle temporal gyrus (MTG) (voxel *p* < 0.005, cluster *p* < 0.05, *GRF* corrected; minimum clusters size of 20 voxels, Figure 2 and Table 2). Additionally, EOS patients showed significantly increased sALFF in the bilateral cerebellum (zone of 4,5) and right caudate nucleus as well as decreased sALFF in the bilateral precuneus (voxel *p* < 0.005, cluster *p* < 0.05, *GRF* corrected; Supplementary Figure S2 and Supplementary Table S1) compared to HCs.

**TABLE 2:** Group differences in dALFF between EOS patients and HCs.

Brain regions	Sphere L/R	Cluster size (voxels)	MNI (mm)			*t*-value
			
			x	y	z	
**Cluster 1**						
Middle temporal gyrus	R	65	66	−42	−3	4.08
**Cluster 2**						
Precuneus	L	95	−6	−72	45	–2.61
Precuneus	R	63	15	−66	30	–2.62
**Cluster 3**						
SupraMarginal gyrus	R	24	60	−15	24	–4.02
						

*dALFF, dynamic amplitude of low-frequency fluctuations; L, left; R, right.*

We further examined the correlations between alterations of dALFF and aALFF in terms of spatial and temporal distribution. We found significant overlap in spatial distribution between dALFF and sALFF in the precuneus and right MGT in EOS patients (Supplementary Figure S5). Moreover, we also found the significant temporal correlations in the MGT and precuneus of EOS patients (Supplementary Figure S6). The findings of other window lengths are shown in Supplementary Figure S7.

### Relationships Between dALFF Variability and Clinical Variables

**FIGURE 3:**
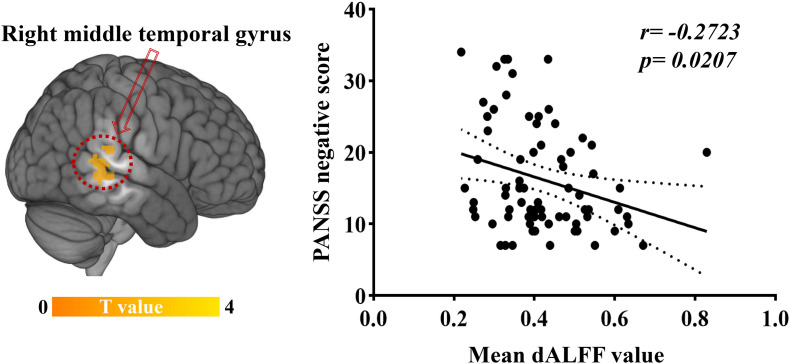
Relationship between dALFF variability and clinical symptoms. Increased dALFF variability of the right MTG was negatively correlated with PANSS negative scores of patients with EOS (*p* < 0.05, uncorrected).

Significant correlations were found between dALFF variability and PANSS scores in EOS patients: the abnormal dALFF variability of the right MTG was negatively associated with PANSS negative scores of EOS patients (*r* = −0.2723, *p* = 0.0207; uncorrected, Figure 3). No significant correlation was found between other abnormal dALFF variability (sALFF) and PANSS scores (Supplementary Figures S3, S4).

## Discussion

In our study, we identified the temporal variability of dynamic spontaneous neural activity in EOS patients by using a novel dALFF method. Enhanced dALFF variability in patients with EOS was prominently located in the right MTG, and was characterized by decreased dALFF variability in the bilateral precuneus, right supramarginal gyrus and right post-central gyrus, which suggests lower neural activity variability in these regions compared to HCs. We also found increased sALFF in the bilateral cerebellum and right caudate nucleus, as well as decreased sALFF in the bilateral precuneus. There was a significant overlap in the spatial distribution between alterations of dALFF and sALFF in the right MTG and precuneus in EOS patients. In addition, enhanced dALFF variability in the right MTG was negatively correlated to negative symptoms of EOS. These findings provide initial evidence to deepen our understand of EOS and highlight the important role of distinguishing between neural patterns in EOS patients and controls.

A primary aim of this study was to identify dALFF variability abnormalities in drug-naïve, first-episode EOS patients. Patients with untreated EOS might directly enhance our understanding of the pathophysiological brain functional activity changes without the confounding effects of antipsychotic medication and disease-related clinical characteristics (Lui et al., 2009). In schizophrenia, the correlations between morphometric measures and neurochemical ones are generally unclear. However, we know that the treatment of diseases mainly intervenes in the neurotransmitter pathways related to schizophrenia through antipsychotic drugs. For example, the dopamine pathway is both a treatment target and a system implicated in the pathogenesis of schizophrenia, while some brain regions such as the medial prefrontal cortex, the striatum and thalamus are within this pathway, therefore, the functional activities of these regions might be affected before and after treatment. Several other areas may also be effected including the parietal and occipital cortex, but they do not receive prominent dopaminergic innervation (Gong et al., 2016).

Functional network characteristics have shown a high sensitivity to antipsychotic medication as well as progressive alterations during the course of illness (Lui et al., 2009). Notably, one previous study found decreased global efficiency in medication-naïve adult schizophrenia patients (Hadley et al., 2014). Together with the current findings, this suggests that alterations of functional activity in untreated patients may represent an early brain-based biomarker for schizophrenia.

dALFF as a neuroimaging measure index that can accurately characterize the brain intrinsic functional tissue at rest, as reflected by the dynamic variability in local neural activity at different sliding windows during the scanning period. EOS patients exhibited increased dALFF variability in the right MTG, which is one of the final regions of development in the brain, and is associated with emotional control (Arsalidou et al., 2011), social cognition (Alcalá-López et al., 2018), moral judgments (Garrigan et al., 2016), and processing complex problems (Christoff et al., 2009). A previous study also found that MTG was correlated to prospective self-referential thinking in schizophrenia (Fornara et al., 2017). Moreover, as a key node of the higher order cognitive network (Owen et al., 2005), dysfunction of MTG and its functional integration is highly correlated to cognitive deficits in adolescents with EOS (Arsalidou et al., 2020).

Cognitive impairments and emotional dysregulation are the most prominent characteristics of schizophrenia and can predict clinical outcomes (Schultz and Andreasen, 1999). MTG hyperactivation may disrupt the capacity to exert emotional control and cognitive regulation in EOS. In our study, EOS patients showed enhanced dALFF variability in the right MTG that was negatively associated with negative symptoms, as well as concurrent dALFF and sALFF abnormalities in this region. These findings suggest that increased neural oscillations and variability are linked to the onset of EOS. Additionally, regions showing enhanced functional activations may be a predominant neural marker for brain maturation at the early stage of schizophrenia (Symond et al., 2005; Uhlhaas and Singer, 2010). In a previous study, we found that abnormal functional integration in the temporal lobe was negatively correlated to negative symptoms of PANSS (Li et al., 2015). This was also found to be associated with auditory processing, language processing (Menon et al., 1995; Bigler et al., 2007), and decreased activation in the temporal lobe during the encoding stage of working memory and language processing in patients with schizophrenia (Haenschel et al., 2007; Simons et al., 2010). In our current study, we observed that increased dALFF variability can better distinguish patients with EOS from HCs and serve as a potential risk element for EOS onset.

We additionally observed regions showing significantly decreased dALFF variability and sALFF in the bilateral precuneus in EOS patients. These findings were consistent with previous static ALFF studies that reported reduced local brain functional activity in the precuneus in patients with adult and adolescents schizophrenia (Hoptman et al., 2010; Zheng et al., 2016). The precuneus is a structural node of the human brain cortex (Hagmann et al., 2008) and plays an important role in the DMN (Friston et al., 2014; Littow et al., 2015), as well as in self-reflection processes, autobiographical memory retrieval, visuo-spatial imagery, and envisioning future events (Buckner et al., 2008; Wang et al., 2019a). Previous studies have found that the dysfunction of the DMN and its altered connection patterns with other brain areas have been associated with positive and negative symptoms in schizophrenia (Bluhm et al., 2007), and that aberrant temporal fluctuations of the DMN might result from an alteration in the connectivity of some core areas with other brain networks in schizophrenia (Garrity et al., 2007). As such, reduced intrinsic brain functional activity of precuneus may contribute to a potential disease-related pathogenesis of schizophrenia and cognitive impairments (Hu et al., 2017).

Moreover, we observed a decreased dALFF variability of the right post-central gyrus, which is consistent with previous fMRI studies showing disrupted local functional integration patterns of the post-central gyrus in schizophrenia (Jiang et al., 2015; Zhao et al., 2018). Previous studies also identified concurrent changes in structural and dynamic functional integration in the supramarginal gyrus, which were both effected by illness and age in EOS patients (Thormodsen et al., 2013; Wang et al., 2019c). Taken together, our findings suggest that changes in dynamic local brain activity in these brain areas may serve as an early biomarker for early EOS diagnosis.

Static ALFF and dALFF exhibited significant spatial overlaps and temporal correlation in the precuneus and MTG in EOS, suggesting that these regions may be significant in the onset and development of EOS. One possible explanation for this is that dALFF is based on sALFF and the sliding window method to explore dynamic brain activity, and the two indicators may be similar in evaluating the physiological mechanisms of brain activity. Unfortunately, we are unable to specifically identify whether dALFF or sALFF is most sensitive to distinguishing patients from HCs. However, brain activity is a dynamically changing process, and we found that increased dALFF variability in the MTG was negatively related to negative symptoms in patients, suggesting that dALFF may be better able to distinguish between EOS patients and HCs, compared to sALFF.

Several limitations should be considered in the current study. First, the length of the sliding window approach is still under contention, and the window length of 50 TR was recommended by previous studies to capture the dynamics of local brain activity. Therefore, other lengths may reveal different pattern results. Second, we recruited participants using a cross-sectional design that does not allow the ability to separate intra- and inter-subject variability. Further longitudinal studies should be conducted to elucidate the neurodevelopmental processes involved in EOS. Third, the GRF method of correction is not necessarily the most powerful correction method, and more rigorous multiple comparison correction methods will be necessary for future studies. Finally, EOS patient brains were registered to the MNI adult brain template, which could result in some age-specific differences in spatial normalization. However, these differences are unlikely to affect the final fMRI results due to their lower spatial resolution (Burgund et al., 2002; Kang et al., 2003; Alexander-Bloch et al., 2010); normalization quality was also monitored by checking the normalization images on a subject-by-subject basis.

## Conclusion

In summary, our study revealed decreased dynamic brain neural activity variability in the precuneus, supramarginal and post-central gyrus, in addition to enhanced dynamic neural variability in the MTG of patients with untreated first-episode EOS. We also identified increased dynamic functional activity variability in the MTG that was negatively related to negative symptoms of EOS. Our findings reveal abnormal dynamic local brain activity at an early stage of the disease and provide insights into identifying the onset and neuropathological progression of EOS.

## Data Availability Statement

The raw data supporting the conclusions of this article will be made available by the authors, without undue reservation.

## Ethics Statement

The studies involving human participants were reviewed and approved by the Ethics Committee of the First Hospital of Shanxi Medical University (Shanxi, China). Written informed consent to participate in this study was provided by the participants’ legal guardian/next of kin.

## Author Contributions

YX designed and supervised this study. QL focused on analysis of experimental data and drafted the manuscript. XC and SL helped to revise the manuscript. ZL, LC, CY, and YW assisted to recruiting participants. All authors discussed the results and commented on the manuscript.

## Conflict of Interest

The authors declare that the research was conducted in the absence of any commercial or financial relationships that could be construed as a potential conflict of interest.

## Footnotes

1 http://rfmri.org/dpabi
