# Supplementary material for: Dynamic Alterations of Amplitude of Low-Frequency Fluctuations in Patients With Drug-Naïve First-Episode Early Onset Schizophrenia
Source: Front Neurosci. 2020 Oct 6;14:901. doi: 10.3389/fnins.2020.00901 (PMC7573348; doi:10.3389/fnins.2020.00901)
Supplement: Supplementary file 1 [file Data_Sheet_1.doc]

**Supplementary Material**

**
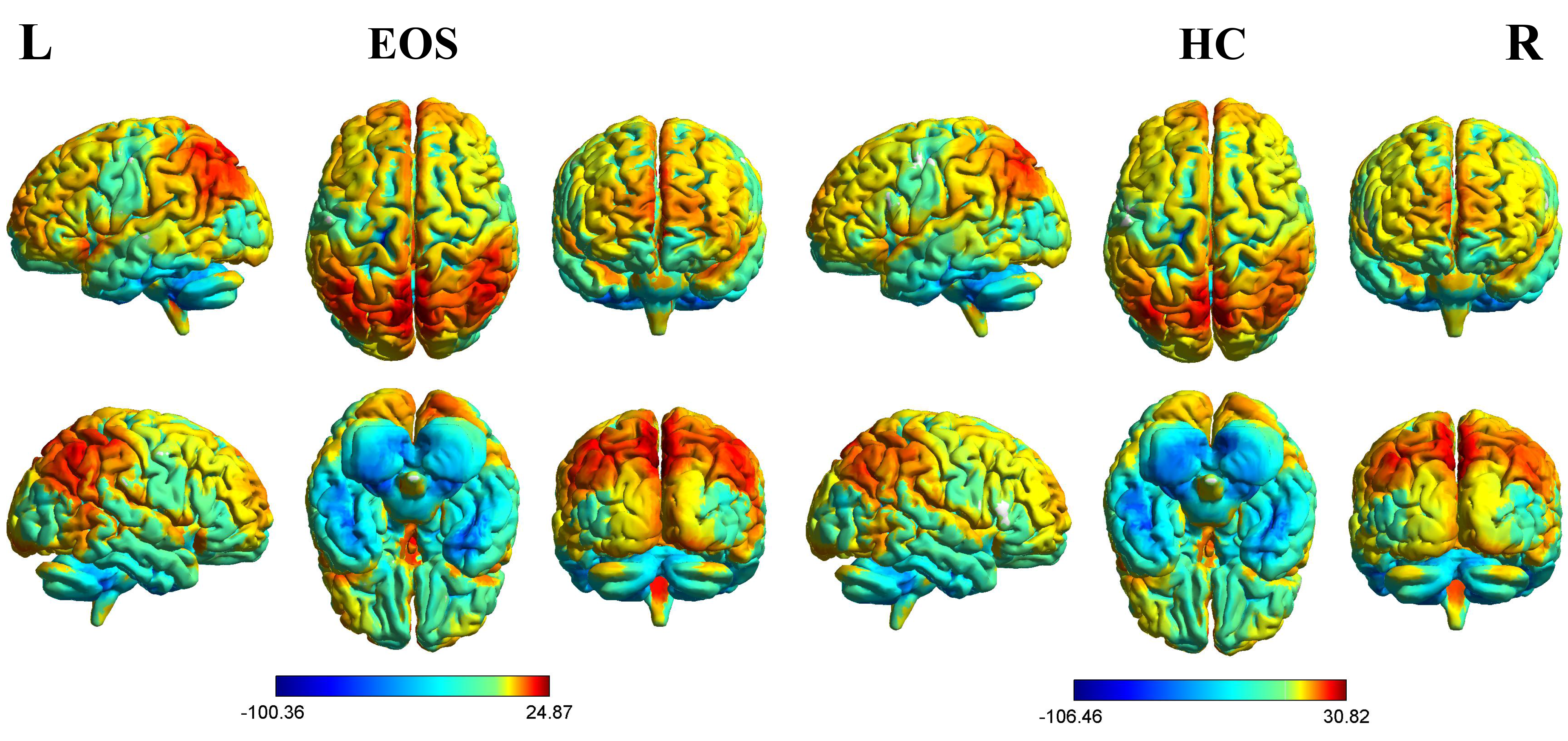
**

**Figure S1. The sALFF distribution in patients with EOS and HCs.** The one sample t tests were applied to reveal the distribution patterns of sALFF in both EOS and HCs (FDR correction; *q* < 0. 05).

**
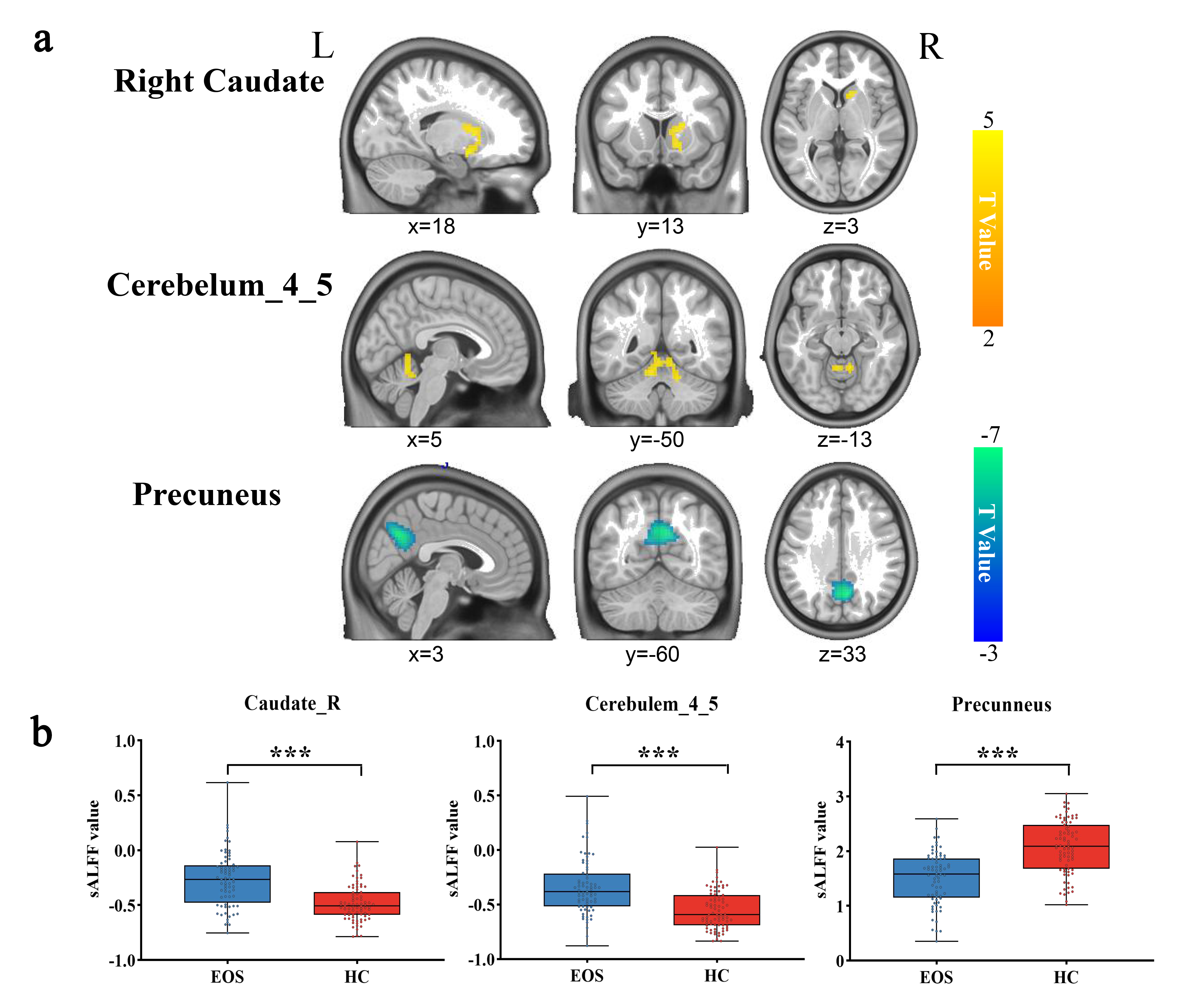
**

**Figure S2. Group differences with sALFF between patients with EOS and HCs.** (a) According to two-sample t-test, patients with EOS exhibited increased sALFF in the bilateral cerebellum (areas of 4 and 5) and right caudate (warm color) and decreased sALFF in the bilateral precuneus (winter color) (GRF corrected; *p* < 0.005; cluster level, *p* < 0.05). (b) The graph exhibiting the ROI-wise post hoc comparison results. **p* < 0.01, uncorrected; ****p* < 0.0001, Bonferroni correction


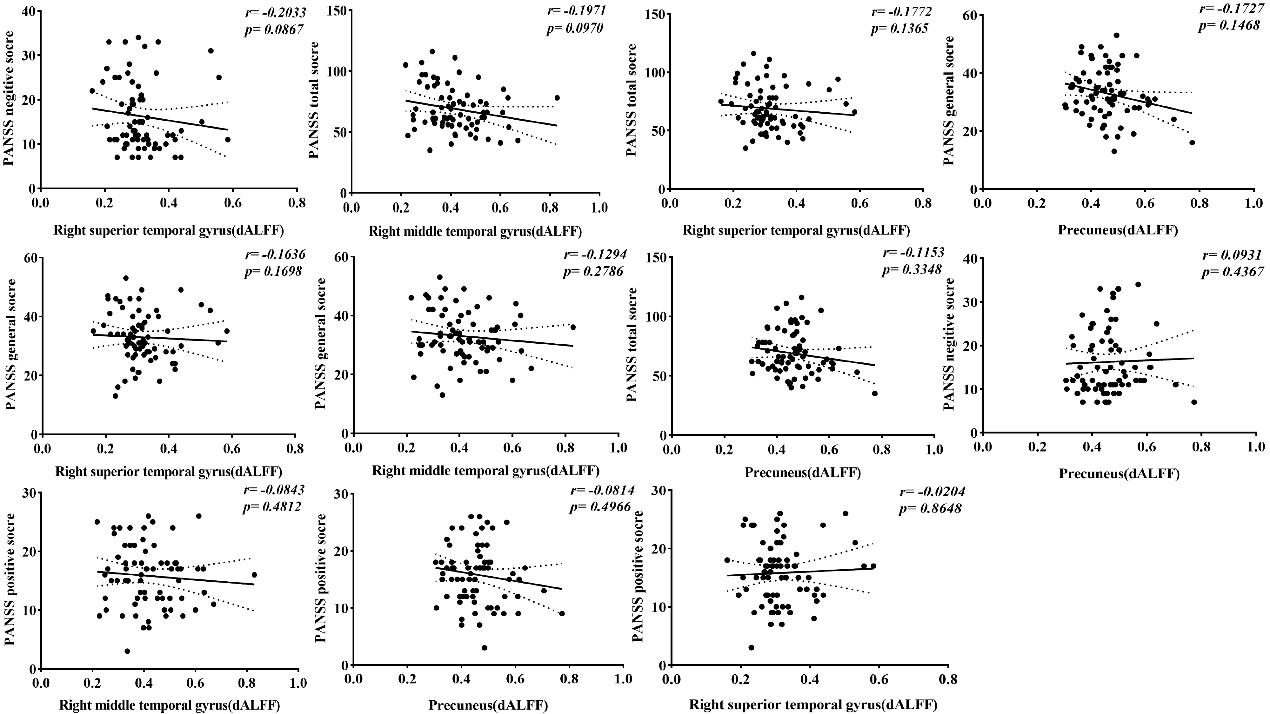


**Figure S3. Correlation analyses between abnormal dALFF variability and PANSS scores.** No significant associations were found between alterations of dALFF variability and PANSS scores in EOS.


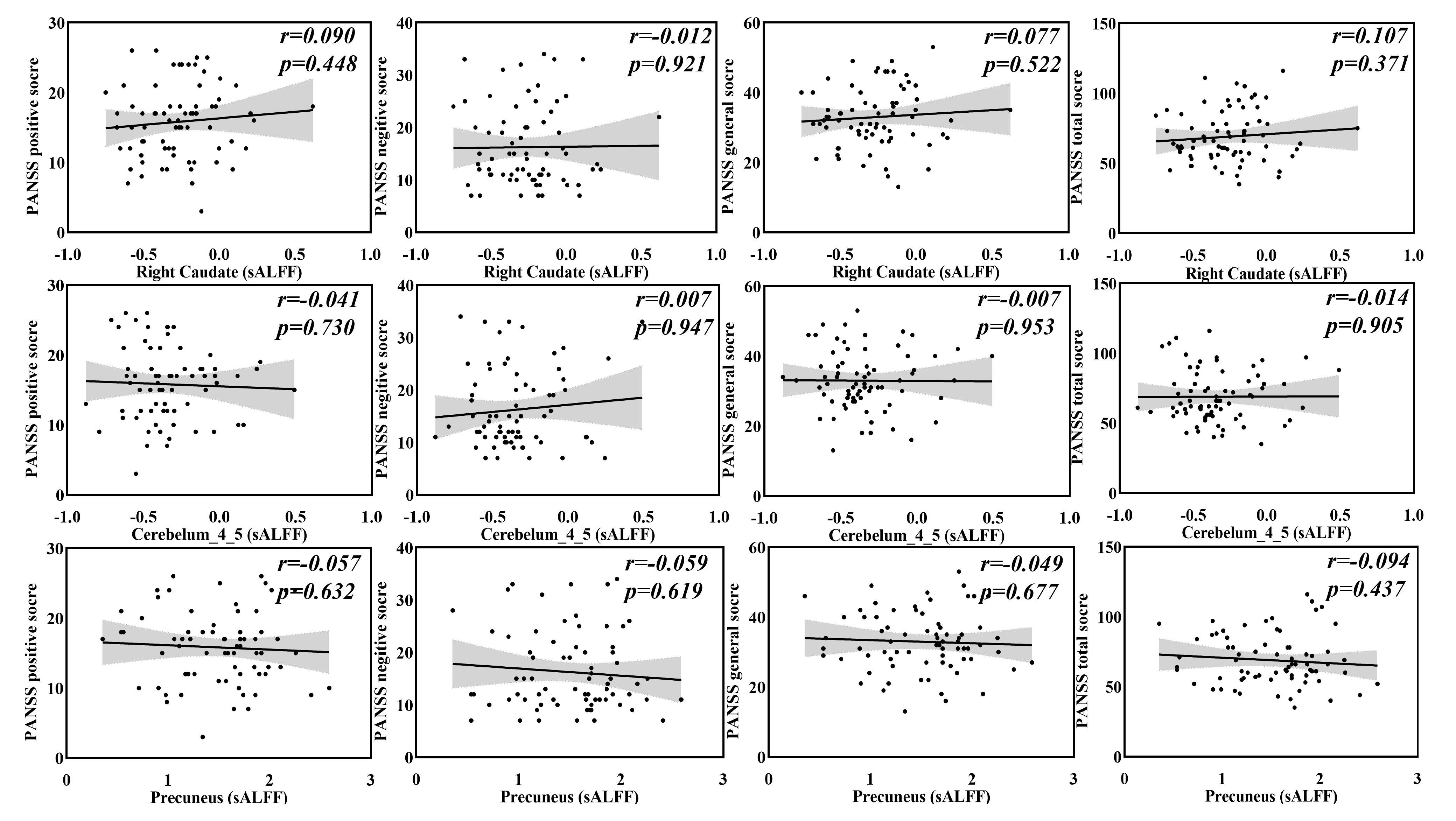


**Figure S4. Correlation analyses between mean sALFF value and PANSS scores.** No significant correlations were found between alterations of sALFF and PANSS scores in EOS.


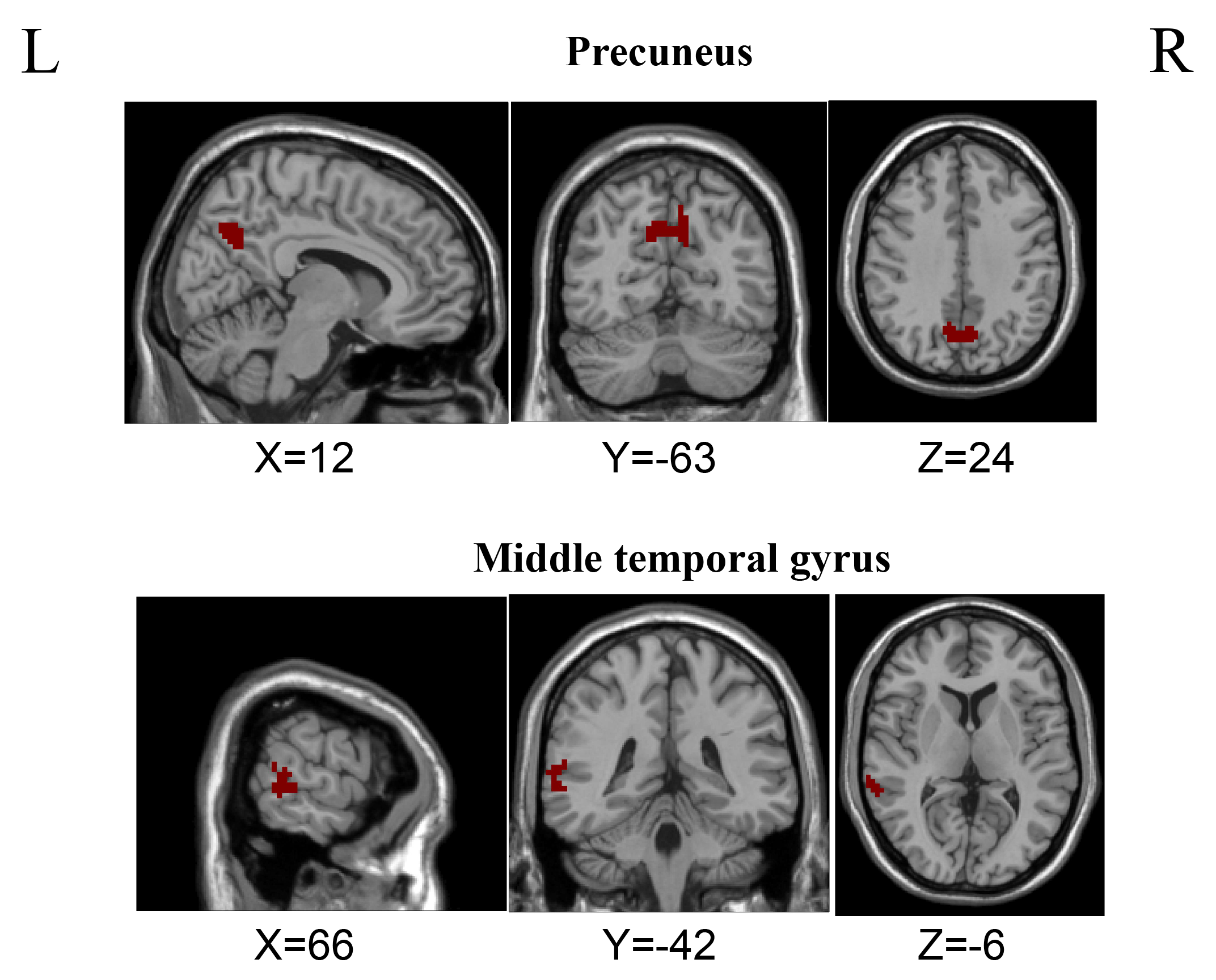


**Figure S5 Associations between alterations of dALFF and sALFF in spatial distribution.** The areas of red indicate where the spatial distribution of sALFF and dALFF overlap, these regions were located in the precuneus (Peak MNI coordinate: 12 -63 24, number of voxels: 123, uncorrected) and right MTG (Peak MNI coordinate: 66 -42 -6, number of voxels: 57) in EOS.

**
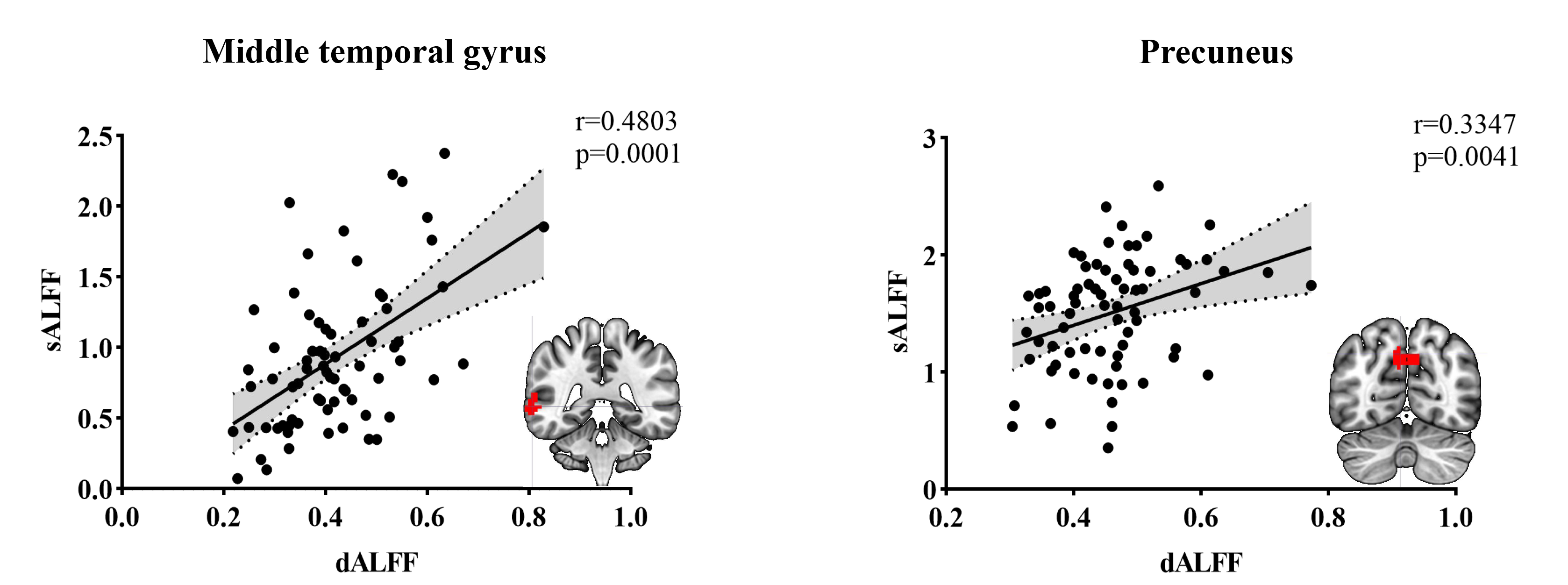
**

**Figure S6 There are significant temporal correlation between dALFF and sALFF in EOS.** There are significant temporal correlations between sALFF and dALFF in right MTG (r= 0.4803, p= 0.0001, uncorrected) and precuneus (r= 0.3347, p= 0.0041, uncorrected) in patients with EOS.


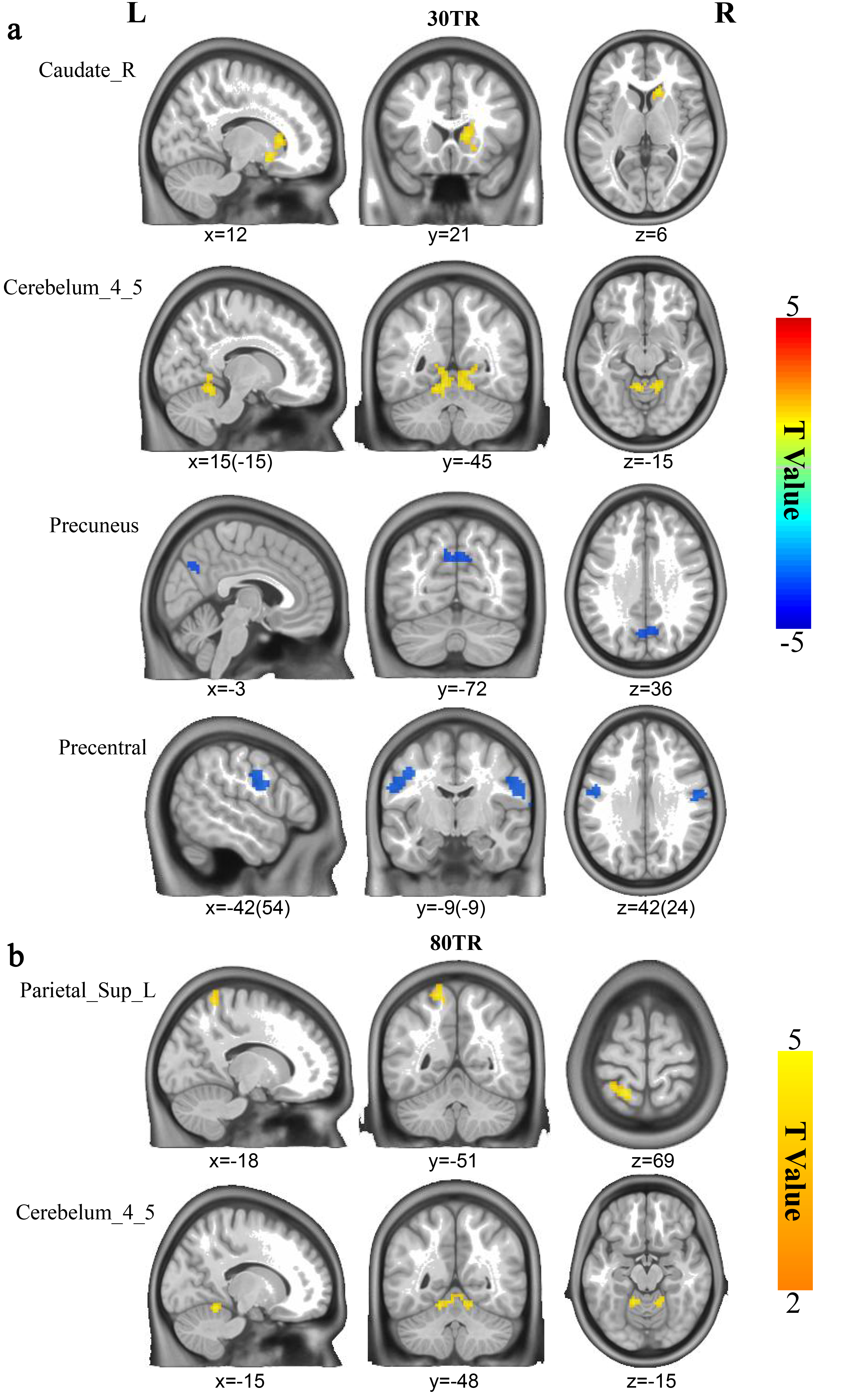


**Figure S7. Additional window lengths of 30TR and 80TR were calculated in dynamic analysis.** (a) When the window length is 30 TR, patients with EOS showed increased dALFF in the bilateral cerebellum and right caudate and decreased dALFF in the bilateral precentral gyrus and precuneus. (b) When the window length is 80 TR, we only found increased dALFF in the bilateral cerebellum and left super parietal gyrus in EOS (GRF corrected; *p* < 0.005; cluster level, *p* < 0.05).

**Table.S1 Group differences in dALFF between EOS patients and HCs.**

| **Brain regions** | **Sphere**  **L/R** | **Cluster size**  **(voxels)** | **MNI (mm)** | | | **t-value** |
| --- | --- | --- | --- | --- | --- | --- |
|  |  |  | **x** | **y** | **z** |  |
| **Cluster 1**  Cerebelum_4_5 | L | 45 | -12 | -51 | -18 | 4.64 |
| Cerebelum_4_5 | R | 36 | 15 | -48 | -24 | 4.96 |
| **Cluster 2**  Caudate | R | 50 | 12 | 15 | 6 | 4.74 |
| **Cluster 3**  Precuneus | L | 153 | 6 | -72 | 36 | -3.52 |
| Precuneus | R | 177 | 6 | -51 | 36 | -3.52 |

Abbreviations: L, left side; R, right side;
